# *Artocarpus altilis* extracts as a food-borne pathogen and oxidation inhibitors: RSM, COSMO RS, and molecular docking approaches

**DOI:** 10.1038/s41598-020-66488-7

**Published:** 2020-06-12

**Authors:** Mohammad Norazmi Ahmad, Nazatul Umira Karim, Erna Normaya, Bijarimi Mat Piah, Anwar Iqbal, Ku Halim Ku Bulat

**Affiliations:** 1 Experimental and Theoretical Research Laboratory, Department of Chemistry, Kulliyyah of Science, International Islamic University Malaysia, 25200 Kuantan, Pahang Malaysia; 2 IUM Poisons Centre, International Islamic University Malaysia, 25200 Kuantan, Pahang Malaysia; 3 Research Unit, IIUM Recreational Park Kuantan Campus, International Islamic University Malaysia, 25200 Kuantan, Pahang Malaysia; 4 Faculty of Chemical & Natural Resources Engineering, Universiti Malaysia Pahang, Lebuhraya Tun Razak, 23600 Gambang Kuantan, Pahang Malaysia; 5 School of Chemical Sciences, Universiti Sains Malaysia, 11800 Penang, Malaysia; 6 Department of Chemistry, Faculty of Science, University Malaysia Terengganu, Mengabang Telipot, 21030 Kuala Terengganu, Terengganu Darul Iman Malaysia

**Keywords:** Natural products, Chemical safety

## Abstract

Lipid oxidation and microbial contamination are the major factors contributing to food deterioration. Food additives like antioxidants and antibacterials can prevent food spoilage by delaying oxidation and preventing the growth of bacteria. *Artocarpus altilis* leaves exhibited biological properties that suggested its use as a new source of natural antioxidant and antimicrobial. Supercritical fluid extraction (SFE) was used to optimize the extraction of bioactive compounds from the leaves using response surface methodology (yield and antioxidant activity). The optimum SFE conditions were 50.5 °C temperature, 3784 psi pressure and 52 min extraction time. Verification test results (Tukey’s test) showed that no significant difference between the expected and experimental DPPH activity and yield value (99%) were found. Gas-chromatography –mass spectrometry (GC-MS) analysis revealed three major bioactive compounds existed in *A. altilis* extract. The extract demonstrated antioxidant and antibacterial properties with 2,3-diphenyl-1-picrylhydrazyl (DPPH) scavenging activity, ferric reducing ability of plasma (FRAP), hydroxyl radical scavenging activity, tyrosinase mushrrom inhibition of 41.5%, 8.15 ± 1.31 (µg of ascorbic acid equivalents), 32%, 37% and inhibition zone diameter of 0.766 ± 0.06 cm (B. cereus) and 1.27 ± 0.12 cm (*E. coli*). Conductor like screening model for real solvents (COSMO RS) was performed to explain the extraction mechanism of the major bioactive compounds during SFE. Molecular electrostatic potential (MEP) shows the probability site of nucleophilic and electrophilic attack during bacterial inhibition. Based on molecular docking study, non-covalent interactions are the main interaction occurring between the major bioactive compounds and bacteria (antibacterial inhibition).

## Introduction

The physical appearance of food is one of the decisive factors in its appeal to consumers and sales of the product. Deterioration of food resulting from chemical and biological means can affect the physical appearance of food and simultaneously, loss of food quality. Chemical processes such as oxidation lead to the degradation of lipids and proteins in food products, whereas biological means, such as microbial contamination, can cause mild to severe foodborne diseases^1^. Therefore, preventing microbial contamination and delaying lipid oxidation are crucial in food production.

As defined by Nimse and Pal^2^, antioxidants are chemical substances that can inhibit or delay the oxidation process, while Meyer *et al*.^3^ defined antimicrobial as an agent that can destroy or inhibit the growth of microorganisms. Antioxidants such as butylated hydroxytoluene (BHT) and butylated hydroxyanisole (BHA) are used to extend the shelf-life of food products by preventing lipid oxidation^1^. However, toxicological effects of synthetic antioxidants have increased interest in natural sources. Natural sources of plant origin are receiving more attention from researchers due to their interesting biological properties and most importantly, it is clear from concerns over halal/haram aspects.

In a previous study, Loizzo *et al*.^1^ proved the antioxidant and antimicrobial activities of the extract of *Artocarpus heterophyllus* leaves. *A. altilis*, which also a *Moraceae* family is believed to have antioxidant and antimicrobial properties. The plant extract with both antioxidant and antimicrobial properties can be a potential replacement for commercial synthetic food preservatives. In this study, *A. altilis* leaves were extracted for use of its phytochemicals as antioxidant and antimicrobial. Formerly, phytochemicals were extracted using a conventional method, which is solvent extraction^4^. However, this method is harmful to human health and the environment. Supercritical fluid extraction (SFE), which is an advanced technology, uses a non-toxic solvent, carbon dioxide (CO_2_) to extract the phytochemicals with high purity and low cost^5^. The efficiency of SFE is dependent on variables such as temperature, pressure, extraction time, modifier and flow rate, which can be optimized using response surface methodology (RSM), a collection of statistical and mathematical techniques^6^.

In this study, the phytochemicals from *A. altilis* leaves were extracted using SFE. RSM was also used to optimize the SFE process. Three variables, temperature (°C), pressure (psi) and extraction time (min), were chosen and analysed using statistical analysis software. Then, the extracts were tested for their antioxidant and antibacterial activities. The chemical compounds in the optimized crude extract were identified using spectroscopic methods. COSMO RS, molecular electrostatic potential (MEP), and molecular docking were performed to study the extraction mechanism, probable sites of nucleophilic and electrophilic attacks, and the interaction between the compounds in the extracts and bacteria, respectively.

## Results and discussion

### Statistical analysis and modelling by RSM

Table 1 shows both the yield and DPPH scavenging activity of the extracts obtained under different testing conditions of SFE. Temperature, pressure and extraction time were used as the independent variables in the SFE process while the yield and DPPH scavenging activity were the dependent variables. Table 2 shows the analysis of variance (ANOVA) results from the evaluation of the quadratic models of yield and DPPH scavenging activity of the extract.

**Table 1:** Experimental Design by RSM.

Run	Variable A Temperature (°C)	Variable B Pressure (psi)	Variable C Extraction time (min)	Response 1 Yield (g)	Response 2 DPPH Scavenging Activity (%)
1	60.00	2200.00	60.00	0.1494	28.9
2	40.00	2200.00	60.00	0.0661	36.3
3	50.00	3300.00	45.00	0.2120	41.5
4	60.00	4400.00	60.00	0.2826	34.1
5	40.00	3300.00	45.00	0.2216	40.9
6	50.00	3300.00	45.00	0.2468	41.8
7	50.00	2200.00	45.00	0.0767	31.3
8	50.00	4400.00	45.00	0.2726	39.3
9	40.00	4400.00	30.00	0.2055	37.2
10	50.00	3300.00	45.00	0.2382	40.4
11	40.00	4400.00	60.00	0.2434	36.8
12	50.00	3300.00	60.00	0.2255	41.2
13	50.00	3300.00	45.00	0.1959	41.9
14	50.00	3300.00	45.00	0.1991	41.3
15	50.00	3300.00	45.00	0.2381	41.3
16	60.00	4400.00	30.00	0.2043	38.3
17	60.00	2200.00	30.00	0.0427	25.0
18	50.00	3300.00	30.00	0.2038	39.2
19	60.00	3300.00	45.00	0.2472	39.1
20	40.00	2200.00	30.00	0.0528	26.4

**Table 2:** ANOVA Results of Yield and DPPH Scavenging Activity of Extract.

	Source of variation	Sum of squares	DF	Mean square	F value	p value
Yield (g)	Model	0.0990	9	0.0110	25.90	<0.0001 significant
	Residual	0.0042	10	0.0004		
	Pure error	0.0024	5	0.0005		
	Lack of fit	0.0018	5	0.0004	0.7384	0.6263 not significant
	Total	0.1032	19			
DPPH scavenging activity (%)	Model	525.66	9	58.41	237.77	<0.0001 significant
	Residual	2.46	10	0.2456		
	Pure error	1.43	5	0.2867		
	Lack of fit	1.02	5	0.2046	0.7138	0.6398 not significant
	Total	528.12	19			

According to Hoe^7^, a *p*-value of less than 0.05 indicates the model is significant. In this study, both of the models show a good fit as the *p*-values are <0.0001 and the lack of fit is not significant (*p* > 0.05). Insignificant lack of fit is good because the fitness of the model is necessary. Potumarthi *et al*.^8^ stated that the closer the *R*^2^ is to 1.0, the better the model predicts the response. In this study, the *R*^2^ value of the DPPH scavenging activity is 0.9953 which is higher than the yield of extract (0.9589). However, both *R*^2^ values of the models are satisfactory because the fitness of the model is signified by the *R*^2^ value of 0.75^9^. Both models recorded smaller adjusted *R*^2^ values than their *R*^2^, which are 0.9219 for yield and 0.9912 for DPPH scavenging activity. The adjusted *R*^2^ value corrects the *R*^2^ value for the sample size and number of terms in the model^8^. Ahmad *et al*.^9^ stated that the adjusted *R*^2^ is smaller than *R*^2^ when there are many terms in the model and the sample size is not large enough.

The adequate precision measures the signal-to-noise ratio, and a ratio of greater than 4 is desirable. The yield and DPPH scavenging activity models give large values of adequate precision, which are 16.6854 and 47.3001, respectively. These large ratios indicate an adequate signal and this suggests that the models can be used to navigate the design space^9^.

The yield presents values of 10.77% and 0.0217 for the coefficient of variation (CV) and prediction errors sum of squares (PRESS). Meanwhile, the CV and PRESS values for DPPH scavenging activity are 1.34% and 19.21, respectively. The low CV value denotes the precision and reliability of the model^8^. As stated by Potumarthi *et al*.^8^, PRESS is a measure of how a particular model fits each point in a design. The smaller the PRESS value, the better the model fits. These models were used for the construction of three-dimensional response surface plots to predict the relationships between independent variables and the dependent variables^6^.

### Effect of the independent variables on the yield of extract

The relationship between the extract yield (Y) and the coded values of independent variables of temperature (A), pressure (B) and extraction time (C) and their interactions are shown in Eq. 1.

1 $$\begin{array}{rcl}{\rm{Y}} & = & 0.2252+0.0137{\rm{A}}+0.0821{\rm{B}}+0.0258{\rm{C}}-0.0044{\rm{AB}}+0.0167{\rm{AC}}\\  &  & -0.0005{\rm{BC}}+0.0038{{\rm{A}}}^{2}-0.0559{{\rm{B}}}^{2}-0.0159{{\rm{C}}}^{2}\end{array}$$

Based on Table 3, the main variables B (pressure) and C (extraction time) are significant whereas variable A (temperature) is not significant because its *p*-value exceeds 0.05^10^. The order of the effect strength of the variables on the response can be determined by comparing the *t*-values. The larger the *t*-value, the stronger the effect of the variables^9^. For the main variables, the ascending order of the effect strength is A < C < B. Based on the *t*-values, it can be concluded that variable B (*t* = 12.565), which is the pressure, affects the yield of extracts more significantly than the extraction time (*t* = 3.938) while the temperature is a non-significant variable towards the yield of extracts.

**Table 3:** ANOVA for the Evaluation of Yield of Extract Regression Model.

Terms	Coefficient estimate	Sum of squares	DF	Mean square	F value	p value	t value
A	0.0137	0.0019	1	0.0019	4.41	0.0621	2.082
B	0.0821	0.0674	1	0.0674	158.68	<0.0001	12.565
C	0.0258	0.0067	1	0.0067	15.67	0.0027	3.938
AB	−0.0044	0.0002	1	0.0002	0.3649	0.5593	−0.586
AC	0.0167	0.0022	1	0.0022	5.27	0.0446	2.310
BC	−0.0005	<0.0001	1	<0.0001	0.0043	0.9493	−0.0480
A^2^	0.0038	<0.0001	1	<0.0001	0.0942	0.7652	0.310
B^2^	10.0559	0.0086	1	0.0086	20.27	0.0011	−4.493
C^2^	−0.0159	0.0007	1	0.0007	1.65	0.2285	−1.277

**Figure 1:**
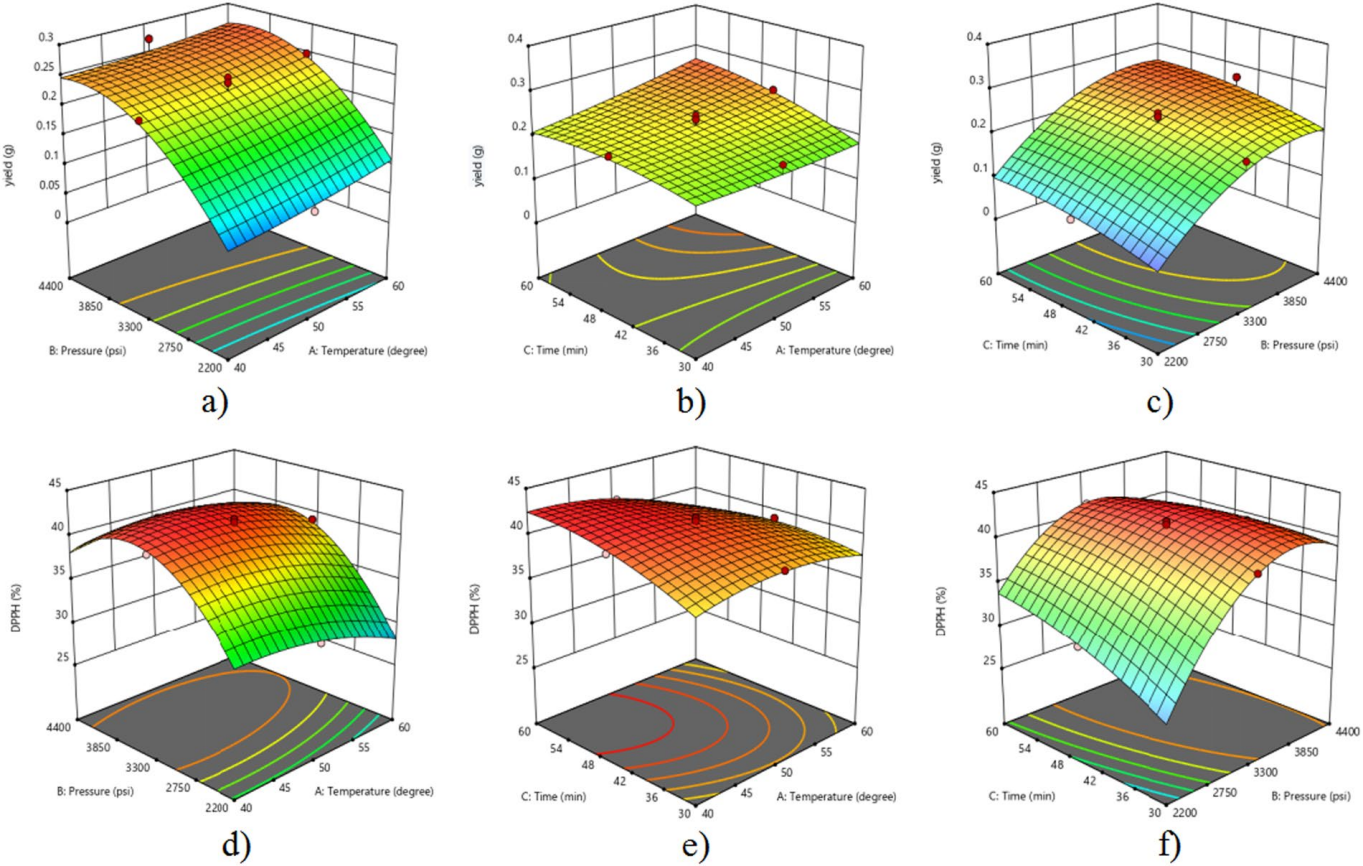
3D model graph of the effect of interaction between (**a**) Temperature and pressure on yield of extract (**b**) Temperature and extraction time on yield of extract (**c**) Pressure and extraction time on yield of extract (**d**) Temperature and pressure on DPPH scavenging activity (**e**) Temperature and extraction time on DPPH scavenging activity (**f**) Pressure and extraction time on DPPH scavenging activity.

Based on Table 3, the interaction effect of temperature and pressure (AB) on the yield of the extract is not significant because it has a *p*-value of 0.5593, which is larger than 0.05. Besides, the *t*-value is small (0.586) indicating that the interaction effect is not strong. In Fig. 1(a), the yield shows a sharp increment with the increase in pressure. This is because when pressure is increased, the density of the supercritical fluid (CO_2_) also increases, resulting in better solubility of compounds of the sample matrix in the solvent^10^. However, the extraction yield of extract was not affected by the increase in temperature. This might be due to degradation of some of the compounds in the extract at high temperature^10^, thus reducing the yield of extract. Moreover, pressure (*t* = 12.565) has a larger *t*-value than temperature (*t* = 2.082), indicating that the effect strength of pressure on the extract yield is larger than temperature. According to Zhang *et al*.^11^, an elliptical contour plot indicates that the interactions between the independent variables are significant. Based on Fig. 1(a), it shows that pressure gives an elliptical shape of contour plot while, for temperature, there is no elliptical shape found.

The interaction effect of temperature and extraction time (AC) is significant based on its *p*-value, which is 0.0446 (Table 3). Moreover, it has a *t*-value of 2.310 (Table 3), which is larger than the interaction of temperature and pressure. This indicates that the interaction effect of temperature and extraction time is stronger than the interaction effect of temperature and pressure. Figure 1(b) shows that there was a slight increase in extract yield when the extraction time is increased. This could be due to the increase in the amount of compounds extracted with the increase in time^12^. However, temperature does not show any significant effect on the yield because the temperature is a non-significant (*p* > 0.05) variable towards the yield of extract. When comparing the *t*-values (Table 2) of both temperature and extraction time, it shows that the *t*-value of extraction time (*t* = 3.938) is larger than of temperature (*t* = 2.082). This indicates the effect of extraction time on the yield of extract is stronger than temperature.

Based on Table 3, the interaction effect of pressure and extraction time (BC) on extract yield gives a *p*-value of 0.9493 and *t*-value of 0.0480. The large *p*-value, exceeding 0.05 indicates that the interaction is not significant while the small *t*-value indicates that the effect of the interaction is not strong^10^. Figure 1(c) shows that the extract yield is increased with the increase of pressure. As stated by Durante, Lenucci, and Mita^13^, the supercritical fluid density will increase if the pressure is increased. The increase of the fluid density resulted in the increase of solubility of compounds to be extracted. On the other hand, the extract yield recorded a slight increase with the increase of time because the amount of compounds extracted is increased^12^. Based on Fig. 1(c), the contour plot of pressure shows a more obvious elliptical shape compared with extraction time which indicates a higher effect strength of pressure (*t* = 12.565) than extraction time (*t* = 3.938), which is also in accordance with their *t*-values.

#### Effect of independent variables on DPPH scavenging activity

The relationship between the DPPH scavenging activity (Y) and the coded values of independent variables of temperature (A), pressure (B) and extraction time (C) and their interactions are shown in Eq. 2.

2 $$\begin{array}{ccc}{\rm{Y}} & = & 41.35-1.22{\rm{A}}+3.78{\rm{B}}+1.12{\rm{C}}+0.900{\rm{A}}{\rm{B}}-1.23{\rm{A}}{\rm{C}}-2.30{\rm{B}}{\rm{C}}\\  &  & {\textstyle }-1.33{{\rm{A}}}^{2}-6.03{{\rm{B}}}^{2}-1.13{{\rm{C}}}^{2}\end{array}$$

Based on Table 4, all three of the independent variables, temperature (A), pressure (B) and extraction time (C), have a significant effect on DPPH scavenging activity as their *p*-values are less than 0.05^7^. The order of effect strength of the variables can be determined by the *t*-values. The higher the value of *t*, the stronger the effect of the variable^13^. For the main variables, the ascending order of the effect strength is C < A < B. Variable B (pressure) with the highest value of *t* which is 24.118 gives the strongest effect on DPPH scavenging activity. Durante *et al*.^14^ stated that pressure plays an important role in the density of the supercritical fluid (CO_2_). The fluid density increases with the increasing pressure, accordingly, increasing the solubility of the compounds to be extracted. Meanwhile, the effect of temperature on the extraction of antioxidants regards the isomerization and degradation of the compounds extracted.

**Table 4:** ANOVA for the Evaluation of DPPH Scavenging Activity Regression Model.

Terms	Coefficient estimate	Sum of squares	DF	Mean square	F value	p value	t value
A	−1.22	14.88	1	14.88	60.59	<0.0001	−7.784
B	3.78	142.88	1	142.88	581.67	<0.0001	24.118
C	1.12	12.54	1	12.54	51.07	<0.0001	7.146
AB	0.90	6.48	1	6.48	26.38	0.0004	5.136
AC	−1.23	12.00	1	12.00	48.87	<0.0001	−6.991
BC	−2.30	42.32	1	42.32	172.28	<0.0001	−13.126
A^2^	−1.33	4.84	1	4.84	19.72	0.0013	−4.441
B^2^	−6.03	99.90	1	99.90	406.69	<0.0001	−20.167
C^2^	−1.13	3.49	1	3.49	14.23	0.0037	−3.772

Based on Table 4, the interaction effect of temperature and pressure (AB) gives values of *p* and *t* of 0.0004 and 5.136, respectively. This indicates that the interaction is significant and the effect strength is strong. Figure 1(d) shows that pressure influences the DPPH scavenging activity more significantly than temperature as it shows a more obvious elliptical contour plot. This is in accordance with the *t*-values recorded in Table 4 in which pressure (*t* = 24.118) gives a higher *t*-value than temperature (*t* = 7.784). This means that DPPH scavenging activity is mainly affected by the fluid density because a change in pressure contributes to a change in fluid density^12^. This result is similar to a previous study by Wang *et al*.^15^ where the antioxidant activity of *Camellia sinensis* was more affected by the pressure than by the temperature. They reported that a higher temperature could accelerate mass transfer and improve the extraction yield. However, the valuable compounds that contribute to antioxidant activity might degrade at high temperature^13^.

According to the *p*-value (<0.0001) in Table 4, the interaction of temperature and extraction time (AC) on DPPH scavenging activity is significant. In addition, the large *t*-value, 6.991 (Table 4) indicates that the interaction effect of temperature and extraction time on DPPH scavenging activity is strong^16^. Fig. 1(e) shows that temperature has a stronger influence on DPPH scavenging activity than extraction time because temperature shows a more elliptical contour plot. This is confirmed with the *t*-values recorded in Table 4 in which temperature (*t* = 7.784) gives a slightly higher value than extraction time (*t* = 7.146). A similar phenomenon was also observed by Mitra *et al*.^16^, where the temperature effect was more significant than the effect of time in the extraction of *Cucurbita maxima* seeds oil.

Based on Table 4, the interaction effect of pressure and extraction time (BC) is significant as the *p*-value is <0.0001 and the effect strength is strong because of the large *t*-value, 13.126. Figure 1(f) shows that pressure affects the DPPH scavenging activity very strongly as the elliptical contour plot is clearly displayed while time only gives a small effect. It can be proven through the *t*-values of pressure and extraction time, in which pressure gives a much higher value 24.118, than extraction time, which only gives a value of 7.146. This result is in accordance with a previous study by Ghasemi, Raofie and Najafi^17^, where extraction time gives less effect than the pressure in the extraction of essential oil from *Myrtus communis* leaves. This result might be due to the fact that the type of compound extracted is not affected by the extraction time^13^.

### Validation of the model

The statistical optimal values of variables were obtained by moving along the major and minor axes of the contour, and the response at the centre point gives the maximum yield of extract and DPPH scavenging activity^11^. The optimum conditions of the SFE achieved with a desirability 0.943 were 50.5 °C temperature, 3784 psi pressure and 52 min extraction time. A validation test was conducted in triplicate at the optimum conditions and the yield of extract and DPPH scavenging activity obtained were compared with the predicted value. A comparison of the predicted (0.26 g of extraction yield and 41.6% DPPH percentage inhibition) and experimental (0.26 g of extraction yield and 41.23% DPPH percentage inhibition) showed a 99% satisfactory agreement and this result was supported with the Tukey’s test (99% not significant difference). This result thus reflects the accuracy and applicability of RSM in optimizing the DPPH activity and yield extraction from *A. altilis*.

### GC-MS

The GC-MS analysis of the *A. altilis* leaf extracts revealed 12 distinct peaks (Supplementary Fig. 1a), which indicate 12 different compounds. The major compounds of *A. altilis* leaf extracts are hexadecanoic acid (40.11%), *cis*-13-octadecenoic acid (20.18%) and cinnamic acid (15.88%) (Supplementary Fig. 1b–d). The GC-MS analysis also show that carboxylic acid is the major compound in the crude extract. Table 5 shows the list of chemical compounds that existed in the *A. altilis* leaf extracts.

**Table 5:** GC-MS Analysis of Artocarpus altilis Leaves Extracts.

Retention Time (min)	Compound ID	Percentage (%)
6.86	Benzenoic acid	0.95
7.92	Benzene (2-methoxyethyl)	0.52
8.83	Hydrocinnamic acid	5.78
9.97	Cinnamic acid	15.88
15.22	Tetradecanoic acid	0.74
21.21	Hexadecanoic acid	40.11
27.43	Cis-13-octadecenoic acid	20.18
28.13	9-octadecenoic	9.81
37.41	Vitamin A	0.92
51.08	17β-hydroxyandrost-4-en-3-onenoic	3.36
58.04	Ursodeoxycholic acid	0.88
64.33	24,25-dihydroxy vitamin D	0.87

### Hydroxyl radical scavenging activity

Hydroxyl radical scavenging activity is the method used to measure the hydroxyl radicals’ production during the initiation stage of the lipid peroxidation process. It functions by withdrawing the hydrogen atom from unsaturated fatty acids (autoxidation) that are commonly found in membranes^14^. The reduction of the hydroxyl radical capacity of the compounds can serve as a significant indicator of the crude extract to acts as a potential antioxidant. In this study, the hydroxyl radical scavenging inhibition rates of the optimized crude extract and ascorbic acid (control) were 32 and 42%, respectively. Based on the results, we can observe that the major compounds such as hexadecanoic, cinnamic and *cis*-13-octadecenoic acid can protect the autoxidation or lipid oxidation by donating an electron to reactive radicals so that they can be converted into stable species.

### Ferric reducing ability of plasma (FRAP) activity

The FRAP assay is a colorimetric reaction based on the ability of an antioxidant to reduce Fe^3+^ to Fe^2+^ in tripyridyltriazine (TPTZ) solution^18^. Fe(III) reduction is an important indicator of the mechanism action of the major compounds (cinnamic acid, hexadecanoic acid and *cis*-13-octadecenoic acid) in the crude extract. The higher the value of FRAP the higher the concentration of complex. In this study, the colour of the test solution changed from yellow to blue and the FRAP concentration value of the optimized crude extract is 8.15 ± 1.31 (µg of ascorbic acid equivalents).

### Mushroom tyrosinase inhibition assay

Mushroom tyrosinase is responsible for the undesirable enzymatic browning of food that takes place during food processing. This enzyme needs to be inhibited to ensure the quality of the food is taken care of. In this study, we have compared the effectiveness between the crude extract and kojic acid (control) towards mushroom tyrosinase inhibition. We obtained that the percentage of mushroom tyrosinase inhibition of the kojic acid is higher than the crude extract, which are 37 and 47%, respectively. This result was supported by Yildizteki *et al*.^19^ findings, where they obtained that the crude extract from *Crocus mathewii* (hexadecanoic acid as one the major compounds) has a lower percentage mushroom tyrosinase inhibition than kojic acid (control).

### MIC and antibacterial activity of the extract

The antibacterial activity of *A. altilis* leaf extract was evaluated against Gram-positive bacteria (B. cereus) and Gram-negative bacteria (*E. coli*) by disc diffusion assay. The MIC value towards *B. cereus* and *E. coli* is same, which is 25 mg/mL of extract. The extract (25 mg/mL) was able to inhibit both *B. cereus* and *E. coli* with inhibition zone diameters of 0.766 ± 0.06 cm and 1.27 ± 0.12 cm, respectively. Meanwhile, the positive control, Streptomycin showed the highest inhibitory activity against both bacteria with inhibition zones diameter of 3.22 ± 0.13 cm for *B. cereus* and 3.00 ± 0.12 cm for *E. coli* (Supplementary Fig. 2).

Agoramoorthy *et al*.^20^ studied the antibacterial activity of the fatty acids methyl esters extract of leaves of *Excoecaria agallocha*. It showed that the extracts were able to inhibit both Gram-positive and Gram-negative bacteria. Therefore, *A. altilis* leaf extracts, which contained 40.11% hexadecanoic acid or palmitic acid, were able to inhibit both Gram-positive and Gram-negative bacteria. In addition to palmitic acid, other major compounds found in *A. altilis* leaf extracts, which are *cis*-13-octadecenoic acid (20.18%) and cinnamic acid (15.88%), also contribute to the antibacterial activity of the extract. This has been proven through previous studies by Jarial *et al*.^21^, Sova^22^ and Heleno *et al*.^23^.

## Computational chemistry

### Optimization of molecular structures

**Figure 2:**
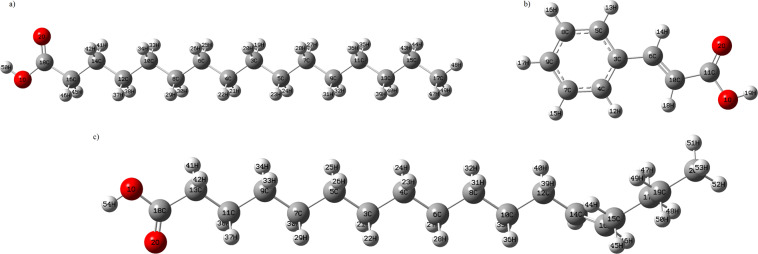
The optimized molecular structure of (**a**) Hexadecanoic acid (**b**) Cis-13-octadecenoic acid and (**c**) Cinnamic acid.

The molecular structures of hexadecanoic acid, *cis*-13-octadecenoic acid and cinnamic acid were optimized using Becke, 3-parameter, Lee–Yang–Parr (B3LYP) 6-311 + +G (d,p) basis set and DFT calculations^24^. The optimized molecular structures constructed using Gauss View 5.0 are shown in Fig. 2. In this study, the molecular structure of hexadecanoic acid, *cis*-13-octadecenoic acid and cinnamic acid were optimized to obtain their ground state energy levels (reactive)^25^. Theoretical data for the bond lengths of the structures calculated using DFT were compared with the experimental data^26^. A correlation graph of experimental against theoretical data was plotted for all three compounds (Supplementary Fig. 3). The correlation coefficient for the bond length of hexadanoic acid (R^2^ = 0.99970), cis-13-octadenoic acid (R^2^ = 0.99978) and cinnamic acid (R^2^ = 0.9980) shows that the results are highly correlated.

### COSMO-RS

In this study, the COSMO-RS approach was used to explain the mechanism of the carbon dioxide in extracting the major bioactive compounds in the *A. altilis* leaves extract. The solubility of the compounds in the solvent can be determined by analysing their sigma profile^27^. The sigma profile is a descriptor that explains the extent of hydrogen bond donor and acceptor within a molecule visualized through a 3D screening charge distribution^28,29^.

**Figure 3:**
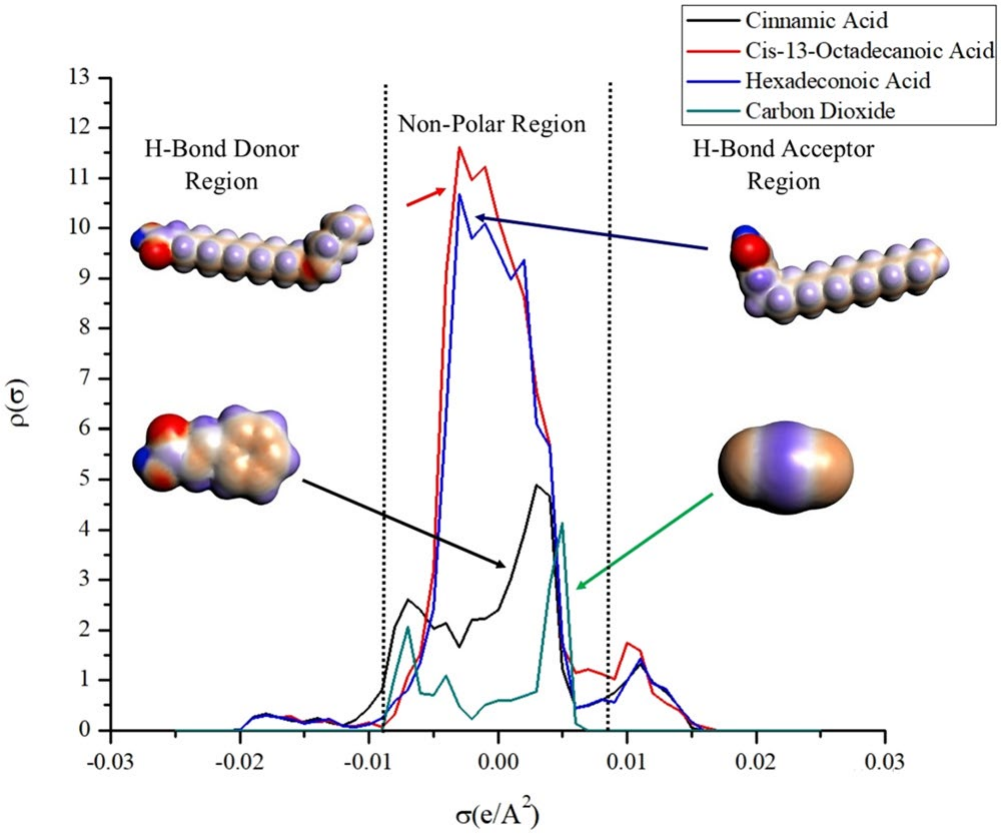
Sigma Profile of of (**a**) Hexadecenoic acid (**b**) Cis-13-octadecenoic acid (**c**) Cinnamic acid (**d**) Carbon dioxide.

**Figure 4:**
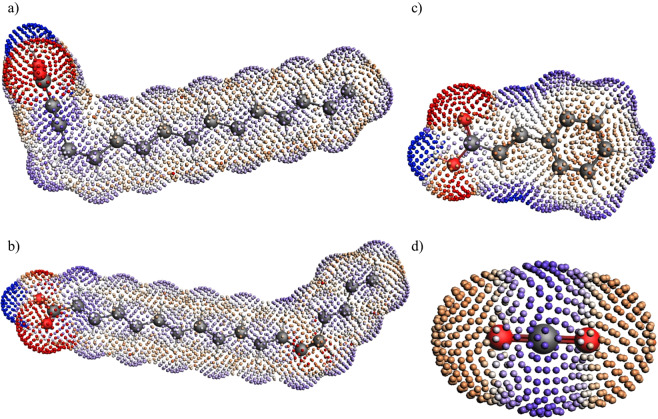
The Surface Charge Density of (**a**) Hexadecenoic acid (**b**) Cis-13-octadecenoic acid (**c**) Cinnamic acid (**d**) Carbon dioxide.

The sigma profile was divided into three significant regions (Fig. 3), which are non-polar, hydrogen bond acceptor and hydrogen bond donor regions^30^. The presence of a peak at the region higher than 0.079 eA^−2^ showed that the molecule has a high tendency to be an electron donor or hydrogen bond acceptor. In contrast, the peak lying below –0.0079 eA^−2^ was associated with electron acceptor or hydrogen bond donor. As can be seen in the sigma profile (Fig. 3), the major bioactive compounds and CO_2_ had significant peaks in the non-polar region (−0.079 eA^−2^ to 0.079 eA^−2^)^31^. The non-polar region is contributed by the polarization of the sp^3^, sp^2^ and sp orbitals of aliphatic group, aromatic group and carbon atom, respectively. Besides that, Fig. 4 also shows the existence of weak hydrogen bond interaction between the oxygen atom in CO_2_ (H bond donor region) and hydrogen atom in major bioactive compounds (H bond acceptor region). This result indicated that hydrophobic interaction is a more significant interaction than hydrogen bonding during the extraction of the targeted compound by carbon dioxide. Thus, it can be stipulated that the solvent (CO_2_), was suitable to be used in the extraction of the targeted compound from the plant matrix because it shared a considerable non-polar property.

### Molecular electrostatic potential (MEP)

**Figure 5:**
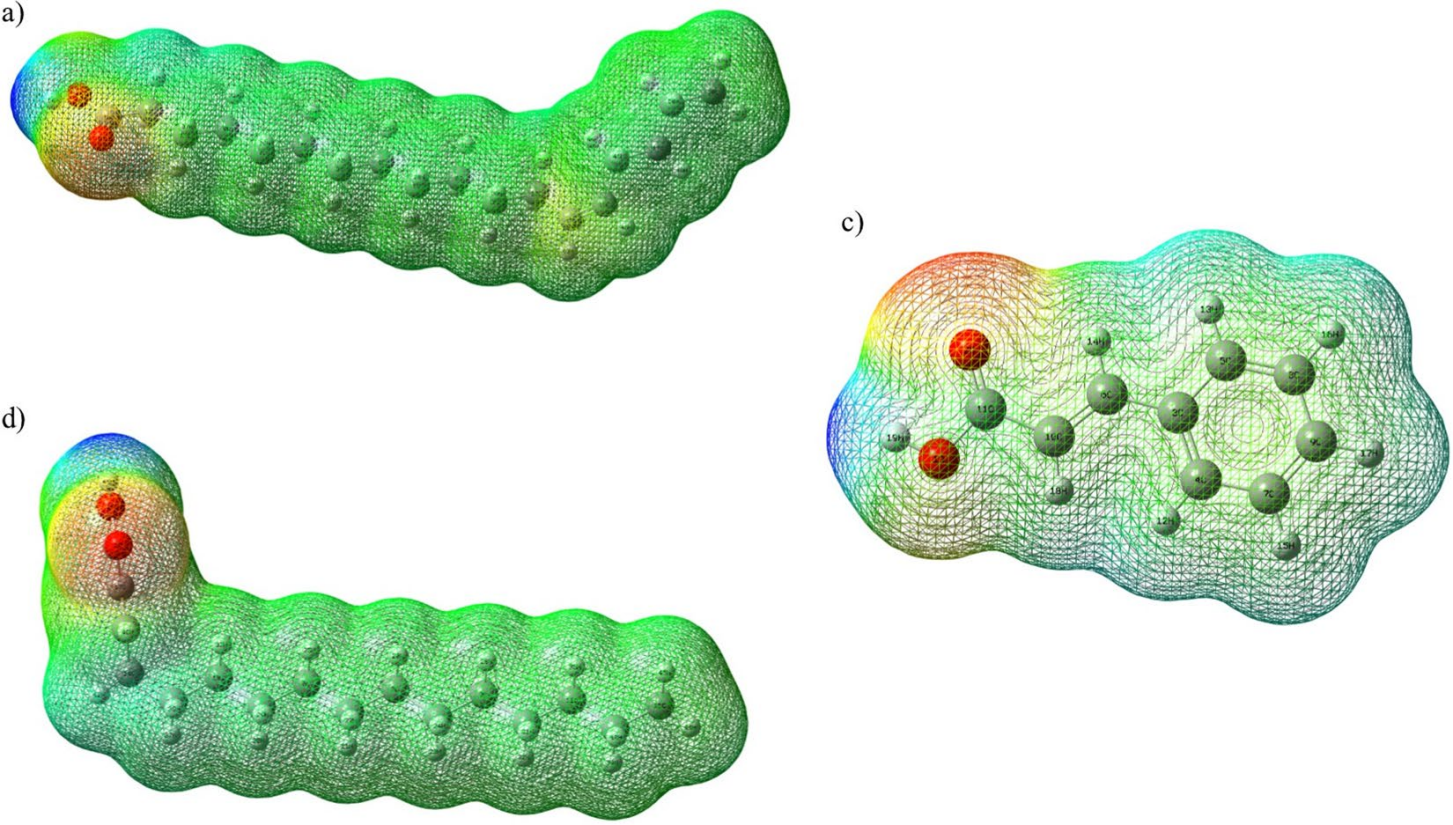
MEP of (**a**) Hexadecanoic acid (**b**) Cis-13-octadecenoic acid and (**c**) Cinnamic acid.

Molecular electrostatic potential (MEP) is the distribution of electrons over the molecule. It has been widely used to visualize the reactivity map that shows the potential region for electrophilic and nucleophilic attack for the chemical interaction (non-covalent and covalent) to occur^32^. In this study, MEP was used to predict the possible interaction site that was involved during the inhibition activity between major bioactive compounds and bacteria^33^. The MEPs of the major bioactive compounds are illustrated in Fig. 5. The surface potentials were represented by five colours with different potential ranked in the following order, blue < green < yellow < orange < red^34^. As can be seen, the deep red spot present over oxygen atoms of carboxylic and phenolic acid groups indicated the nucleophilic attack region. Meanwhile, the deep blue region present over the hydrogen atom indicated the electrophilic attack region. Basically, it represents the highest potential region for the interaction of the major bioactive compounds with protein receptors of *E. coli* and *B. cereus*. Thus, it was predicted that this region is the most probable site for the interaction (non-covalent and covalent) to occur during bacterial activity inhibition.

### Molecular docking studies

The complex data files of the bacteria (*B. cereus* and *E. coli*) and tyrosinase protein were obtained from the Protein Data Bank. PC-PLC (Bc), *E. coli* pyruvate dehydrogenase and tyrosinase from *Agaricus bisporus* as the protein receptors were prepared by removing the water and co-crystallized ligand using Discovery Studio Visualizer. Molecular docking was used to model the interaction between the compounds and a protein receptor^35,36^. According to Churchill and Wetmore^37^, the interactions commonly occurring between biomolecules are non-covalent interactions, namely, van der Waals, hydrogen bond, hydrophobic, charge transfer and π–π interactions.

**Figure 6:**
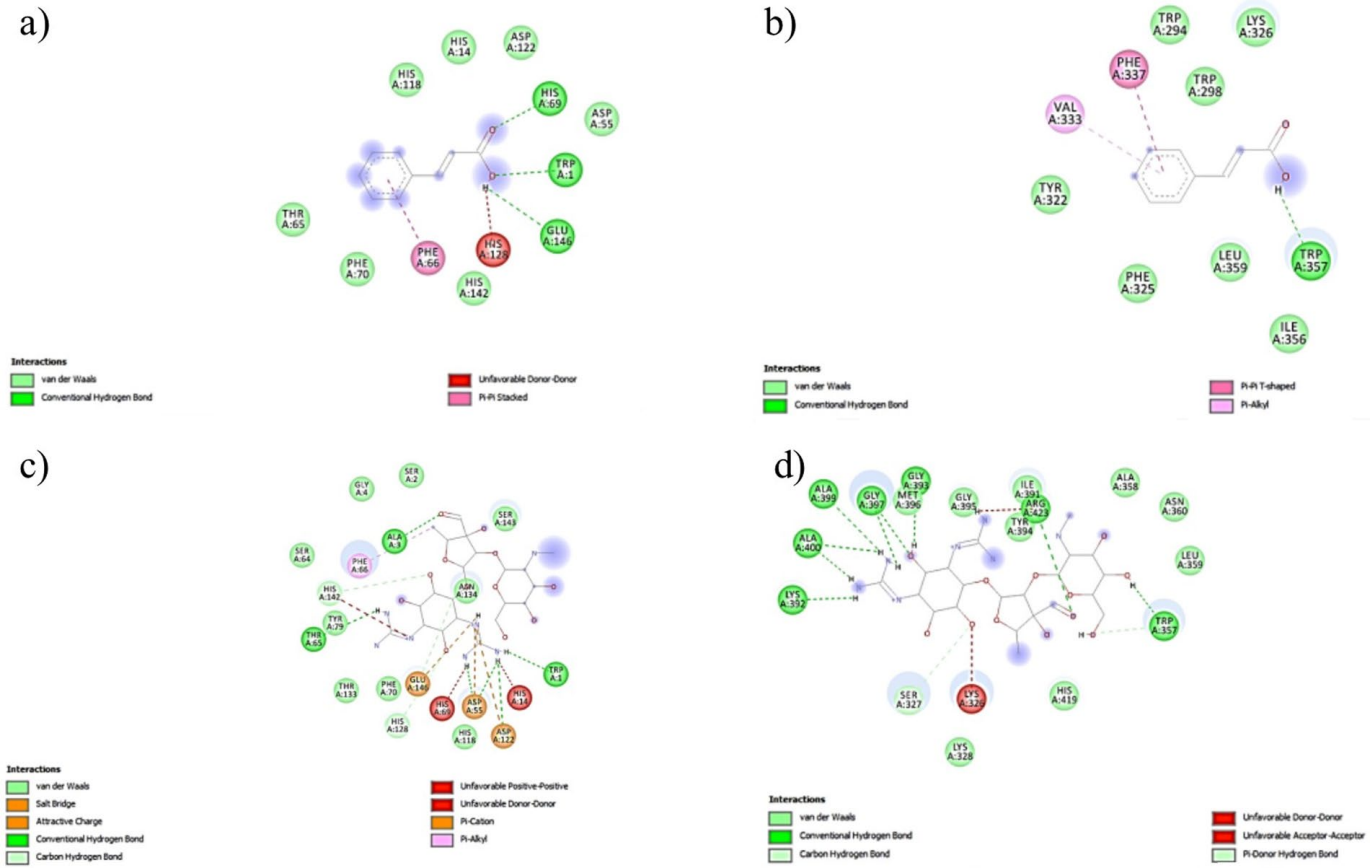
Two-dimensional interaction of (**a**) Cinnamic acid with amino acid residues of PC-PLC (Bc) (**b**) Cinnamic acid with amino acid residues of E.coli pyruvate dehydrogenase (**c**) Streptomycin with amino acid residues of PC-PLC (Bc) (**d**) Streptomycin with amino acid residues of *E. coli* pyruvate dehydrogenase.

**Figure 7:**
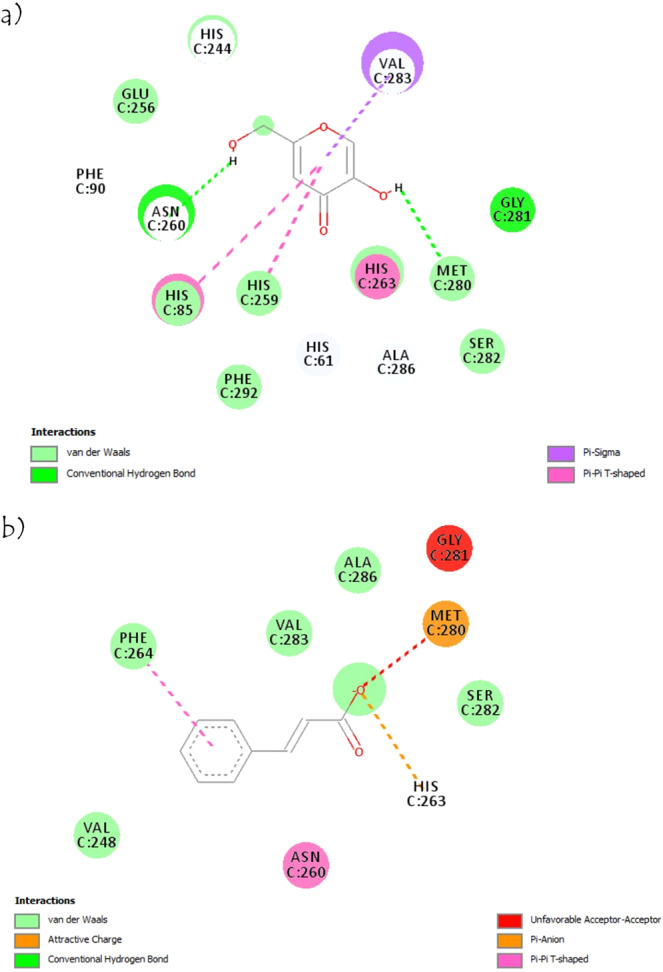
Two-dimensional interaction of (**a**) Kojic acid with amino acid residues of tyrosinase (**b**) Cinnamic acid with amino acid residues of tyrosinase.

Figures 6(a–d) and 7(a,b) show the non-covalent interactions that contribute to the bacterial and enzyme inhibitions between cinnamic acid (the highest binding affinity among other compounds in the crude extract) with *E. coli*, *B. cereus*, and tyrosinase mushroom, respectively. Both of the Streptomycin and kojic acid were used as a control for the bacterial and tyrosinase inhibition, respectively. The effect of the bioactive compounds on the bacteria can be divided into two groups, namely, *E. coli* and *B. cereus*. The order for both the *B. cereus* and *E. coli* were the same, namely, Streptomycin (−7.2, −8.7 kcal/mol) > cinnamic acid (−6.4, −7.3 kcal/mol) > *cis*-13-octadecenoic acid (−4.7, −6.9 kcal/mol) > hexadecanoic acid (−4.7, −6.7 kcal/mol). While the ascending order for the tyrosinase mushrrom inhibition are kojic acid (−5.6 kcal/mol) > cinnamic acid (−5.4 kcal/mol)> *cis*-13-octadecenoic acid (−5.2 kcal/mol) > hexadecanoic acid (−3.5 kcal/mol). The binding affinity is related to the number and types of bonds (hydrogen bond, Van der Waals and η-bond) formed between the bioactive compounds and *B. cereus* and *E. coli*. The hydrogen bond has the highest binding strength^38^ followed by η-bond (interaction between aromatic rings)^39^ and Van der Waals^38^. The higher the total number of stronger bonds, the higher the binding affinities of the interaction (Supplementary Tables 1 and 2). This result is also aligned with the inhibition zone diameter recorded in the antibacterial activity study and tyrosinase mushroom inhibition assay section, where Streptomycin as the control inhibits *B. cereus* and *E. coli* better than the extract with a larger inhibition zone diameter, and kojic acid has a higher percentage tyrosinase inhibition.

## Conclusions

The optimum variables to acquire the maximum yield and DPPH scavenging activity of the extract were 3784 psi pressure, 50.5 °C and 52 min extraction time. GC-MS revealed that *Artocarpus altilis* leaves contain 3 major compounds that possess antioxidant and antibacterial activity, such as hexadecanoic acid, *cis*-13-octadecenoic acid and cinnamic acid. Their antioxidant activity has been proven by their ability to scavenge DPPH radical, hydroxyl radical, reduce Fe^3+^ to Fe^2+^ by 41.5%, 32% and 8.15 (µg of ascorbic acid equivalent), respectively at the optimum condition of SFE. In addition, optimized crude extract also shows the inhibition (37%) on the mushrrom tyrosinase. Based on antibacterial activity, crude extract was successfully *B. cereus* and *E. coli* growth with inhibition zone diameters of 0.766 ± 0.06 cm and 1.27 ± 0.12 cm, respectively. COSMO RS calculation explain the extraction mechanism of the major bioactive compound by using SFE. MEP shows the probability of the interaction occurred between the compounds with both bacteria and these were supported and visualized by a molecular docking technique.

## Materials and methods

### Materials

Ethanol (99.8%) and 2,2-diphenyl-1-picrylhydrazyl (DPPH) were purchased from Sigma-Aldrich (Malaysia). Nutrient Agar (NA), Nutrient Broth (NB) and streptomycin were obtained from Oxied. A Gram-positive bacterium, *Bacillus cereus* (ATCC 11778) and a Gram-negative bacterium, *Escherichia coli* (ATCC 25922) were used in the experiment. All reagents and solvents used were of analytical reagent grade.

### Sample preparation

*A. altilis* leaves were collected at Kuantan, Pahang, Malaysia. Then the leaves were placed in a sealed plastic bag and dried in a freeze dryer at −30 °C. The dried leaves were blended by using waring blender (USA) to form a powder (mean particle size = 0.3 μm). The mean particle size of the powder was determined by using model Mastersizer 2000, brand Malvern (United Kindom) (Supplementary Fig. 4)^40^.

### Extraction of bioactive compounds

Twenty g samples of *Artocarpus altilis* leaves powders were placed in the extraction vessel of SFT-150 SFE system (Supercritical Fluids Extraction, Inc, USA). The pressure was adjusted at the back-pressure regulator and solvent pumps. The flow rate for CO_2_ was fixed at 5 mL/min. Once the set temperature and pressure were achieved after turning on the injection valve and the system was at equilibrium, the extraction was carried out. The extract was collected in a flask connected to the back-pressure regulator, weighed and stored at 4 °C before further analysis^6^.

### Optimization of extraction on yield and antioxidant activity

A central composite design (CCD) was employed for optimization of the process variables (pressure, temperature and extraction time). For each variable, a conventional level was set to zero as the coded level^9^. Antioxidant activity was assumed to be under the influence of the three variables tested. Three different levels (low, medium and high) of each variable were studied. Table 1 shows the treatment combinations of the variables when using RSM. Upon completion of the RSM experiments, a second-order polynomial equation was fitted to the data by multiple regression procedures^9^. The data were then analysed using statistical software package Design Expert (Design‐Expert 11, New York). For a three-factor system, the second-order model equation was evaluated as follows (Eq. 3):

3 $${\rm{Y}}={{\rm{X}}}_{0}+{{\rm{X}}}_{{\rm{a}}}{\rm{A}}+{{\rm{X}}}_{{\rm{b}}}{\rm{B}}+{{\rm{X}}}_{{\rm{c}}}{\rm{C}}+{{\rm{X}}}_{{\rm{ab}}}{\rm{AB}}+{{\rm{X}}}_{{\rm{ac}}}{\rm{AC}}+{{\rm{X}}}_{{\rm{bc}}}{\rm{BC}}+{{\rm{X}}}_{{\rm{aa}}}{{\rm{A}}}^{2}+{{\rm{X}}}_{{\rm{bb}}}{{\rm{B}}}^{2}+{{\rm{X}}}_{{\rm{cc}}}{{\rm{C}}}^{2}$$

where,

Y = predicted response,

X_0_ = intercept,

X_a_, X_b_, X_c_ = coefficient estimates of linear terms,

X_ab_, X_ac_, X_bc_ = coefficient estimates of interaction terms,

X_aa_, X_bb_, X_cc_ = coefficient estimates of quadratic terms,

A = Temperature, B = Pressure and C = Extraction time.

### Verification at the optimum level

A verification test was conducted at optimum levels of the optimized variables as obtained by RSM. The experiment was conducted in triplicate and the DPPH activity and extraction yield were determined to validate the previously predicted optimized conditions. Tukey’s test was used to compare between the means by using OriginPro software. The experimental value of the response was measured under the optimal recommended conditions of the extraction and was compared with the predicted value by means of replicate determination (*n* = 3) in order to determine the validity of the model. Results were considered statistically significant at p ≤ 0.05.

### Gas chromatography-mass spectrometry (GC-MS)

The optimized crude extracts from the leaves of *A. altilis* were analysed using a PerkinElmer GC-MS. The inert gas, helium, was used as a carrier gas at a constant flow rate of 2 mL/min. Detector and injector temperatures were maintained at 250 °C. The initial oven temperature was set at 45 °C for 0.5 min, ramped at 10 °C/min until 150 °C and held for 1.0 min. Finally raised to 250 °C at 4 °C/min and held for 20.0 min. The sample was diluted with ethanol. The diluted sample (1 μL) was taken in a syringe and injected into the injector. The percentage compositions of the crude extract constituents were expressed as a percentage by peak area. The chemical components of the crude extract were determined by comparison of their GC retention indices and mass spectra with those reported in the literature and the National Institute of Standards and Technology (2017) library^41^. In addition, all the major compounds were validated using authentic compounds (hexadecanoic acid, *cis*-13-octadecanoic acid and cinnamic acid) using the same condition above^42,43^.

### Antioxidant test by DPPH assay

The antioxidant activity of the extract was determined by using DPPH. Briefly, 2 mL of 200 μg/mL of the extract was mixed with 2 mL of 0.5 mM ethanolic solution of DPPH. The mixture was shaken and allowed to stand at room temperature for 30 min. Then, the absorbance was recorded at 517 nm using a UV-Vis spectrometer. A lower absorbance of the sample indicated a higher DPPH scavenging activity. The antioxidant activity of the extract was expressed as DPPH scavenging activity in percentage as Eq. 4 below^6^:

4 $${\rm{DPPH}}\,{\rm{scavenging}}\,{\rm{activity}}=({{\rm{A}}}_{0}-{{\rm{A}}}_{{\rm{i}}}/{{\rm{A}}}_{0})\times 100$$

where,

A_0_ = Absorbance of DPPH as control,

A_i_ = Absorbance of DPPH in the presence of sample

### Antioxidant test by hydroxyl radical scavenging activity

Hydroxyl radical (•OH) scavenging activity was measured based on Cai *et al*.^18^ method with some modification. Briefly, the reaction mixture was prepared from the combination of 1 mL of 7 mM ferrous sulfate, 1 mL of 9 mM salicylic acid–ethanol and 7 mL of 200 µM of crude extract. Then, 1 mL of 9 mM hydrogen peroxide was added to the reaction mixture, incubated at 37 °C for 30 min and its absorbance was measured at 510 nm. The hydroxyl radical scavenging activity was calculated according to Eq. 5 below:

5 $${\rm{Scavenging}}\,{\rm{activity}}\,( \% )=[{{\rm{A}}}_{0}-({{\rm{A}}}_{1}-{{\rm{A}}}_{2})]/{{\rm{A}}}_{0}\times 100$$

where, *A*_0_, *A*_1_ and *A*_2_ are the absorbances of the control (DPPH solution without sample), solution mixture of the DPPH and sample, and sample solution without DPPH, respectively. The experiment was performed in triplicate.

### Antioxidant test by ferric reducing or antioxidant power assay (FRAP)

The total antioxidant power of the crude extract was assayed according to the Benzie and Strain^18^ method with minor modification. The results are expressed as ascorbic acid equivalents (mmol/mL) or FRAP units.

### Mushroom tyrosinase inhibition assay

Mushroom tyrosinase inhibition assay was conducted based on the Lan *et al*.^44^ method with some modification. A mixture of 2 mM l-tyrosine (80 µL, Sigma, USA), potassium phosphate buffer (50 mM, pH 6.5) and crude extract (100 µL) were prepared in 96-well plates and incubated for 10 min at room temperature. Then, 20 µL of mushroom tyrosinase (1000 Units/mL, Sigma, USA) was added and incubated for another 30 min. The reaction was stopped by addition of 0.1 N HCl to the reaction mixture before the absorbance was measured at 490 nm. The percentage of mushroom tyrosinase inhibition was calculated based on Eq. 6:

6 $${\rm{Percentage}}\,{\rm{Inhibition}}\,( \% )=[1-({\rm{S}}-{\rm{SB}})/({\rm{C}}-{\rm{CB}})]\times 100 \% $$

where S, SB, C and CB are the absorbances of the sample, the blank sample, the control, respectively.

## Antibacterial test

### Preparation of bacteria

The stock cultures of Gram-positive bacteria (*Bacillus cereus* - ATCC 11778) and Gram-negative bacteria (*Escherichia coli* - ATCC 25922) were grown on NA at 37 °C for 24 hours. Then, the sub-culturing process tooke place to get single colonies. After 24 hours of the incubation period, three well-isolated colonies of the same morphological type were selected from an agar plate culture. The top of each colony was touched with a loop and transferred into a falcon tube containing 10 mL of NB. The bacteria were incubated at room temperature for 48 hours. Then, the optical density (OD) of the incubated bacteria was measured using a Lamda 35 UV-Vis spectrometer (Perkin Elmer, Boston, Massachusetts, USA)^1^.

### Minimum inhibitory concentration (MIC)

The MIC of the crude extract was determined using the microdilution method using 96-well microplates^45^. The crude extract was prepared in ethanol to make 200 mg/L as a stock solution. This concentration was then serially diluted and transferred to the broth media in a 96-well microplate to obtain 5, 25, 50, 100 and 200 mg/mL. Then, 100 μL inoculum (108 CFU/mL for *E. coli* and *Staphylococcus aureus* was added to each well and incubated at 37 °C for 24 h. In this study, broth containing Streptomycin was used as a positive control.

### Disc diffusion

Blank discs were impregnated with 10 μL of 25 mg/mL of extracts and left to dry for a few minutes. Then, the discs were placed on the agar surface, which had been pre-inoculated with the suspension of bacteria (adjusted to 0.08–0.1 McFarland standard) and incubated for 24 hours at 37 °C. Ethanol was used as the negative control and Streptomycin as the positive control. After the incubation period, the plates were observed and the diameters of the inhibition zones (mm) were measured. All tests were performed in triplicate^1^.

## Computational chemistry

### DFT calculation

Density functional theory (DFT) calculations were carried out using the Gaussian 09 program to optimize the chemical structure of compounds to be analysed. Three-dimensional structures of the compounds (ID: 985, 5312441, 444539) were taken from the PubChem. DFT/B3LYP at the 6–311 + +G (d,p) basis set level was adopted to optimize geometrical parameters in the gas phase and the most stable conformation was taken from the final optimization step calculation for all theoretical considerations. The Gauss View 5.0 program was used to construct the optimized molecular structure. Then, the optimized structures were used in molecular docking and generating the molecular electrostatic potential (MEP)^46^.

### COSMO-RS calculation

The structures of the major bioactive compounds (hexadecanoic acid, *cis*-13-octadecenoic acid, and cinnamic acid) and the solvent (carbon dioxide) were obtained from PubChem (CID: 71308850, 5312441, 44539 and 280, respectively). Then, the continuum solvation COSMO calculations and geometries for both solvent and titled compound were optimized using DFT calculations with the Becke–Perdew-86 (BP86) functional and triple zeta valence potential (TZVP) basis set. COSMO files containing the ideal screening charges on the molecular surface were generated. All the quantum chemical calculations were carried out using the Amsterdam Density Functional (ADF) program package, version 2017^11^.

### Molecular docking

Three-dimensional structures bacteria (ID: 2HUC, 1L8A), tyrosinase (ID: 2Y9X) and compounds to be analysed were taken from the DFT calculation section and Protein Data Bank (PDB) database. The ADT 4.2 software was used to calculate the binding affinity between the protein receptor and the analysed compounds. The interaction was analysed using Discovery Studio Visualizer 2016^24^.

## Supplementary information

Supplementary Information .
