# Author Correction: *Artocarpus altilis* extracts as a food-borne pathogen and oxidation inhibitors: RSM, COSMO RS, and molecular docking approaches

**DOI:** 10.1038/s41598-020-76567-4

**Published:** 2020-11-05

**Authors:** Mohammad Norazmi Ahmad, Nazatul Umira Karim, Erna Normaya, Bijarimi Mat Piah, Anwar Iqbal, Ku Halim Ku Bulat

**Affiliations:** 1 Experimental and Theoretical Research Laboratory, Department of Chemistry, International Islamic University Malaysia, 25200 Kuantan, Pahang Malaysia; 2 IIUM Poisons Centre, International Islamic University Malaysia, 25200 Kuantan, Pahang Malaysia; 3 Research Unit, IIUM Recreational Park Kuantan Campus, International Islamic University Malaysia, 25200 Kuantan, Pahang Malaysia; 4 Faculty of Chemical & Natural Resources Engineering, Universiti Malaysia Pahang, Lebuhraya Tun Razak, 23600 Gambang Kuantan, Pahang Malaysia; 5 School of Chemical Sciences, Universiti Sains Malaysia, 11800 Penang, Malaysia; 6 Department of Chemistry, Faculty of Science, University Malaysia Terengganu, Mengabang Telipot, 21030 Kuala Terengganu, Terengganu Darul Iman Malaysia

Correction to: *Scientific Reports* 10.1038/s41598-020-66488-7, published online 12 June 2020

The original version of this Article contained an error in Affiliation 2, which was incorrectly given as ‘IUM Poisons Centre, International Islamic University Malaysia, 25200, Kuantan, Pahang, Malaysia’. The correct affiliation is listed below:

IIUM Poisons Centre, International Islamic University Malaysia, 25200, Kuantan, Pahang, Malaysia.

This error has now been corrected in the HTML and PDF versions of the Article, and in the accompanying Supplemental Material.

