# Supplementary material for: Artocarpus altilis extracts as a food-borne pathogen and oxidation inhibitors: RSM, COSMO RS, and molecular docking approaches
Source: Sci Rep. 2020 Jun 12;10:9566. doi: 10.1038/s41598-020-66488-7 (PMC7293230; doi:10.1038/s41598-020-66488-7)
Supplement: Supplementary file 1 — Supplementary Information . [file 41598_2020_66488_MOESM1_ESM.pdf]

Supplementary Information

Artocarpus altilis extracts as a food-borne pathogen and oxidation inhibitors: RSM, COSMO RS, and molecular docking approaches

Mohammad Norazmi Ahmad,[1,2] Nazatul Umira Karim,[1] Erna Normaya Abdullah,[1,3] Bijarimi Mat Piah,[4] Mohammad Anwar Mohamed Iqbal,[5] Ku Halim Ku Bulat[6]

[1]Experimental and Theoretical Research Laboratory, Department of Chemistry, Kulliyyah of Science, International Islamic University Malaysia, 25200 Kuantan, Pahang, Malaysia.

[2] IUM Poisons Centre, International Islamic University Malaysia, 25200 Kuantan, Pahang, Malaysia.

[3]Research Unit, IIUM Recreational Park Kuantan Campus, International Islamic University Malaysia, 25200 Kuantan, Pahang, Malaysia.

[4]Faculty of Chemical & Natural Resources Engineering, Universiti Malaysia Pahang, Kuantan, Malaysia

[5] School of Chemical Sciences, Universiti Sains Malaysia, 11800 Penang, Malaysia.

[6]Department of Chemistry, Faculty of Science, University Malaysia Terengganu, Mengabang Telipot 21030 Kuala Terengganu**,** Terengganu Darul Iman, Malaysia

Correspondence and requests for materials should be addressed to Ahmad, MN (Email: mnorazmi85@gmail.com or mnorazmi@iium.edu.my)

List of Supplementary of Figure Legends

Supplementary Fig. 1	Chromatogram of the a) crude extract from *A. Altilis* b) Cinnamic acid, c) Hexadecnoic acid, and d) Cis-Octadecanoic acid.
Supplementary Fig. 2	Effect of *A. Altilis* extracts and Streptomycin (25 mg/mL) agains a) Bacillus Cereus b) Escherichia Coli using Disc Diffusion method.
Supplementary Fig. 3	The bond length correlation coefficient of a) Hexadecanoic acid b) cis-13-octadecanoic acid and c) Cinnamic acid.
Supplementary Fig. 4	The mean particle size of the *A. Altilis* powder.

Supplementary Figures

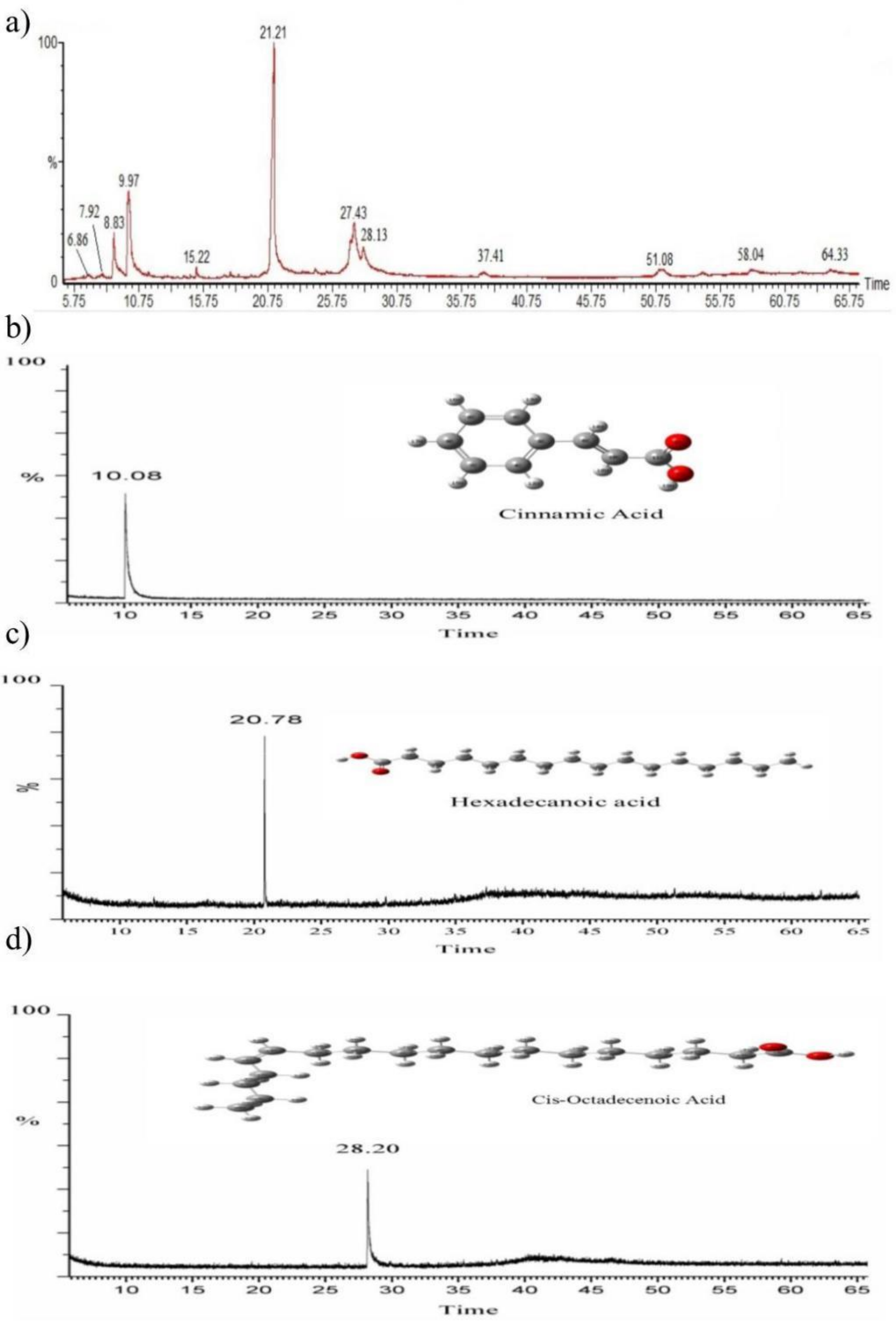

Supplementary Fig. 1 GC-MS Chromatogram of *Artocarpus altilis*

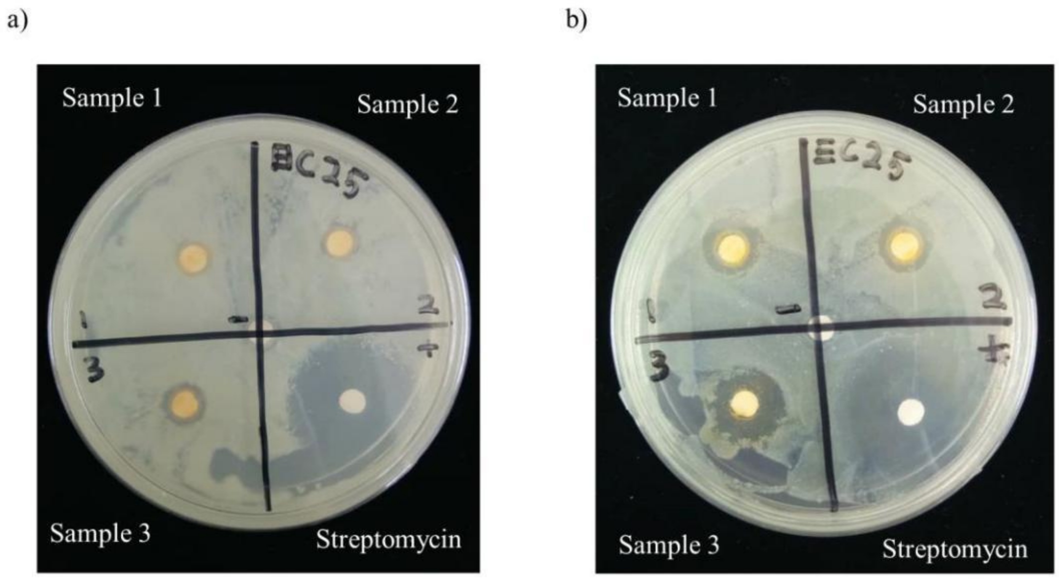

Supplementary Fig. 2 Effect of *Artocarpus Altillis* extracts and streptomycin (200 mg/mL) against a) *Bacillus Cereus* b) *Escherichia Coli* using Disc Diffusion Method.

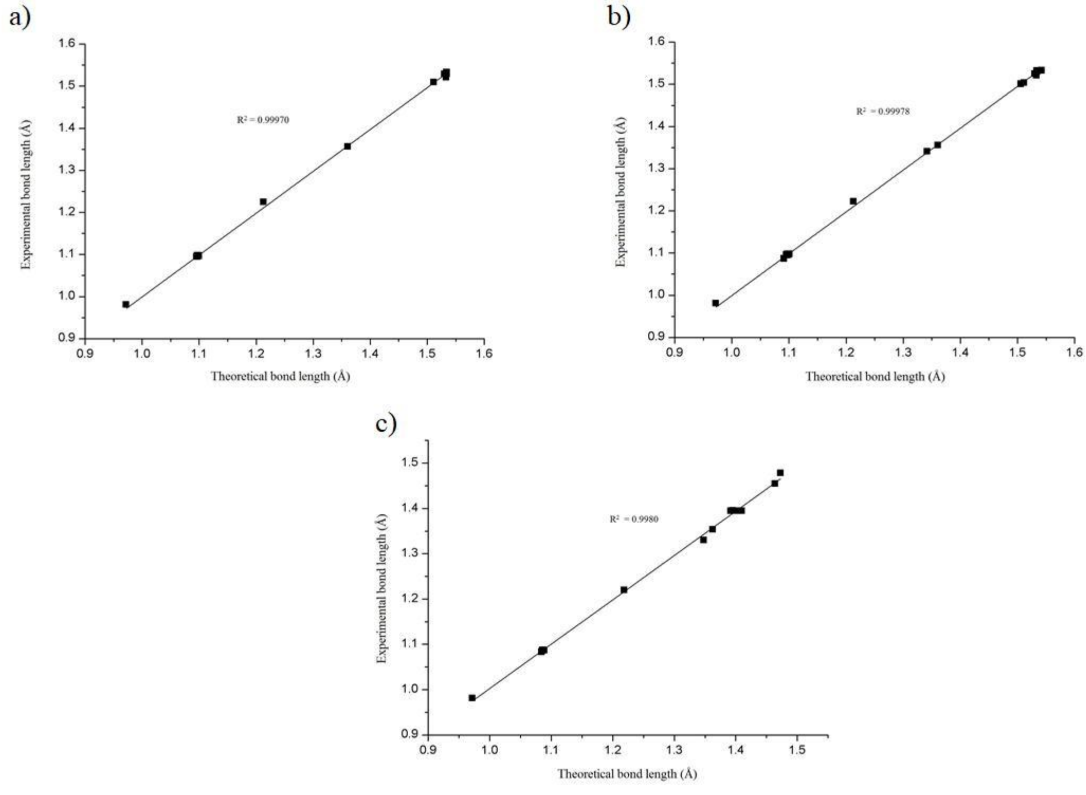

Supplementary Fig. 3 The bond length correlation coefficient of a) Hexadecanoic acid b) Cis-13-octadecenoic acid and c) Cinnamic acid.

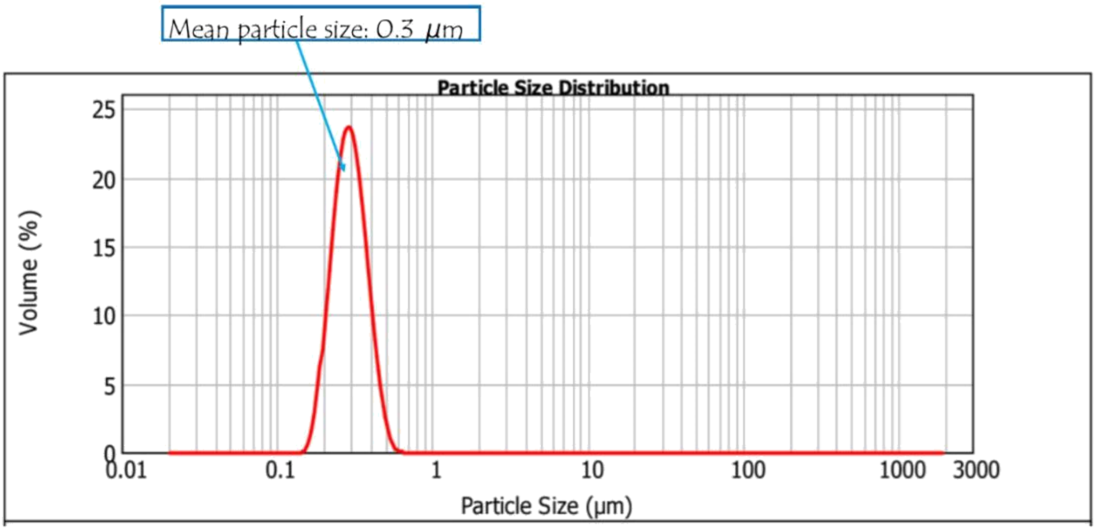

Supplementary Fig. 4 The mean particle size of the *A. altilis* powder.

List of Supplementary Tables

Supplementary Table 1: The Binding Affinity Values and Number of Bonds Formed in Hexadecanoic Acid, Cis-13-Octadecenoic Acid, Cinnamic Acid and Streptomycin (Control)

Supplementary Table 2 The Binding Affinity Values and Number of Bonds Formed in Hexadecanoic Acid, Cis-13-Octadecenoic Acid, Cinnamic Acid and Streptomycin (Control) and Tyrosinase

Supplementary Tables

Supplementary Table 1 The Binding Affinity Values and Number of Bonds Formed between Hexadecanoic Acid, Cis-13-Octadecenoic Acid, Cinnamic Acid, Streptomycin (Control) and Bacteria.

No	Compounds	Bacteria	Affinity (kcal/mol)	Number of Bonds formed		
				Hydrogen Bond	Van der Waals	η Bond
1	Hexadecanoic Acid	*B. cereus*.	-4.7	0	9	1
		E. coli	-6.7	2	11	2
2	Cis-13-Octadecenoic Acid	*B. cereus*.	-4.7	0	12	1
		E. coli	-6.9	0	14	2
3	Cinnamic Acid	*B. cereus*.	-6.4	3	7	1
		E. coli	-7.3	1	7	2
4	Streptomycin (Control)	*B. cereus*.	-7.2	10	4	5
		E. coli	-8.7	12	4	0

Supplementary Table 2 The Binding Affinity Values and Number of Bonds Formed in Hexadecanoic Acid, Cis-13-Octadecenoic Acid, Cinnamic Acid and Streptomycin (Control) and Tyrosinase

No	Compounds	Enzyme	Affinity (kcal/mol)	Number of Bonds formed		
				Hydrogen Bond	Van der Waals	Other bonds
1	Hexadecanoic Acid	tyrosinase	-3.5	2	6	0
2	Cis-13-Octadecenoic Acid	tyrosinase	-5.2	2	6	1
3	Cinnamic Acid	tyrosinase	-5.4	2	6	3
4	Kojic Acid (Control)	tyrosinase	-5.6	2	8	3
